# Deformable Polyurethane Joints and Fibre Grids for Resilient Seismic Performance of Reinforced Concrete Frames with Orthoblock Brick Infills

**DOI:** 10.3390/polym12122869

**Published:** 2020-11-30

**Authors:** Theodoros Rousakis, Alper Ilki, Arkadiusz Kwiecien, Alberto Viskovic, Matija Gams, Petra Triller, Bahman Ghiassi, Andrea Benedetti, Zoran Rakicevic, Camilla Colla, Omer Faruk Halici, Bogusław Zając, Łukasz Hojdys, Piotr Krajewski, Fabio Rizzo, Vachan Vanian, Anastasios Sapalidis, Efthimia Papadouli, Aleksandra Bogdanovic

**Affiliations:** 1 Civil Engineering, Democritus University of Thrace, 67100 Xanthi, Greece; vachvani@civil.duth.gr (V.V.); anassapa@civil.duth.gr (A.S.); evthpapa11@civil.duth.gr (E.P.); 2 Civil Engineering Department, Istanbul Technical University, Istanbul 34469, Turkey; ailki@itu.edu.tr (A.I.); halici@itu.edu.tr (O.F.H.); 3 Faculty of Civil Engineering, Cracow University of Technology, 31-155 Cracow, Poland; akwiecie@pk.edu.pl (A.K.); bozajac@pk.edu.pl (B.Z.); lukasz.hojdys@pk.edu.pl (Ł.H.); piotr.krajewski@pk.edu.pl (P.K.); 4 Engineering and Geological Department, Gabriele D’Annunzio University of Chieti-Pescara, 65122 Pescara, Italy; alberto.viskovic@tin.it (A.V.); fabio.rizzo@unich.it (F.R.); 5 Department of Structural and Earthquake Engineering, University of Ljubljana, 1000 Ljubljana, Slovenia; matijagams@gmail.com; 6 Slovenian National Building and Civil Engineering Institute (ZAG), The Department of Structures, 1000 Ljubljana, Slovenia; petra.triller@zag.si; 7 Department of Civil Engineering, University of Nottingham, Nottingham NG7 2RD, UK; bahmanghiassi@gmail.com; 8 Department of Civil, Chemical, Environmental, and Materials Engineering, University of Bologna, 40126 Bologna, Italy; andrea.benedetti@unibo.it (A.B.); camilla.colla@unibo.it (C.C.); 9 Institute of Earthquake Engineering and Engineering Seismology (IZIIS), Ss. Cyril and Methodius University in Skopje, 1000 Skopje, North Macedonia; zoran_r@iziis.ukim.edu.mk (Z.R.); saska@iziis.ukim.edu.mk (A.B.)

**Keywords:** polyurethane, flexible joint, RC column, brick infill, shake table, resilience

## Abstract

The behaviour of reinforced concrete frames with masonry wall infills is influenced a lot by the stiffness and strength difference between the frame and the infill, causing early detrimental damage to the infill or to the critical concrete columns. The paper reports the results from shake table seismic tests on a full-scale reinforced concrete (RC) frame building with modified hollow clay block (orthoblock brick) infill walls, within INMASPOL SERA Horizon 2020 project. The building received innovative resilient protection using Polyurethane Flexible Joints (PUFJs) made of polyurethane resin (PU), applied at the frame-infill interface in different schemes. Further, PUs were used for bonding of glass fibre grids to the weak masonry substrate to form Fibre Reinforced Polyurethanes (FRPUs) as an emergency repair intervention. The test results showed enhancement in the in-plane and out-of-plane infill performance under seismic excitations. The results confirmed remarkable delay of significant infill damages at very high RC frame inter-story drifts as a consequence of the use of PUFJs. Further, the PUFJ protection enabled the resilient repair of the infill even after very high inter-story drift of the structure up to 3.7%. The applied glass FRPU system efficiently protected the damaged infills against collapse under out-of-plane excitation while they restored large part of their in-plane stiffness.

## 1. Introduction

Brittle materials (e.g., clay bricks, concrete [1]) can be found in the majority of civil engineering buildings and especially in those that are made of masonry or reinforced concrete (RC). Connectors of brittle structural members may be: stiff joints (capable to carry high loads but unable to undergo large deformations) or sealants (capable to undertake high deformations but unable to carry high loads) [2]. There appears to be a gap between the two cases in construction industry. Thus, a new sort of structural joint has been introduced, named Polymer Flexible Joints (PFJs) [3]. The main advantages of the PFJ come from the hyperelastic characteristics of the special polyurethanes forming flexible joints [4,5]. The joints can carry high loads and undergo large deformations. Moreover, the stress concentrations in joints are reduced by redistributing stresses over a large bond area [5,6,7,8,9]. Structures made of brittle materials located in seismic areas [2,10] and repaired with stiff joints may show high vulnerability. Micro-cracks form inside brittle materials where high stress concentrations occur [8]. The stiffer is the joint between two brittle structural elements, the higher will be the peaks of stress concentration, responsible for micro cracks formation and thus weakening of brittle materials [4,10]. Typically, cracks are repaired using stiff, brittle mineral or epoxy grouts, which do not improve significantly the structural capacity (damage energy) [2,3,11,12], because of stress concentrations and low deformability. Strengthening of masonry and concrete structures using composite materials and stiff adhesives is not fully effective because of the low strength of the substrates [7,13,14,15]. Stress concentrations overcome strength of the substrates, generated by stiff adhesives [12,16,17,18,19]. In seismic areas, high amount of ductility and deformation capacity of structures are required [1,5,20,21], thus other bonding solutions are sought, more compatible with masonry and concrete substrate [22,23,24,25]. The PFJ introduces greater tensile and shear resistance, deformability and ductility and thus greater bearing capacity (damage energy) in the bonded brittle structural elements, also in the post failure deformation zone—making the structure safer [1,15]. PFJ can repair cracks by injection or filling structural gaps [2,3,10,11,23,24], to make flexible hinges (using polyurethanes of PM and PT type)—polyurethane flexible joints (PUFJs), as well as bonding of composites using flexible adhesives (PS and PT) [4,6,7,12,13,14,15]—fibre reinforced polyurethanes (FRPUs). Polyurethanes of various stiffness and deformability (e.g., polymers: PT—stiff, PS—middle stiff and PM—soft) have to be designed properly in the PFJ to assure the compatibility with various substrates under variable loading conditions. Various technologies based on the PFJ were investigated in laboratory and in-situ tests and their practical effectiveness was manifested in several civil engineering applications [2,3,10,21]. However, the application of this technology in infilled RC frames under seismic actions and its efficiency in improving the structural performance has not been addressed yet.

Several past studies investigated the effects of different intervention techniques on infill walls to improve their shear strength and displacement ductility, to provide infill-walled frames with desirable and reliable inelastic behaviour (sprayable ductile fibre reinforced cementitious composites [26]). Fibre reinforced polymer sheet strengthening has also been investigated widely [27,28,29,30]. There were tested [31] old-type deficient RC frames with infill walls strengthened with textile reinforced mortar. In most of the cases under cyclic loading, the retrofitted infill walls showed failure mechanism of limited ductility and behaviour similar to the non-retrofitted ones, despite the enhancement of their shear resistance and of the dissipated energy [32]. There was severe accumulation of damage in the range of 0.5–1.5% lateral drift, limiting the potential of the retrofitted RC frames [31]. This aspect is crucial to the desirable ductile behaviour of retrofitted infill walled frames to avoid uncertain effects of the infill walls that may lead to soft-story mechanism [33] and potential whole-building collapses in cases of seismic induced overloads or out of plane collapse of infills. Several analytical studies of retrofitted structures have revealed the beneficial effects of the suitable infill wall retrofitting scheme with polyurethane FRP (FRPU) and novel techniques [34,35,36,37,38,39]. Strengthening with flexible polymers enables engaging all different infills in different places and levels and raises the shear capacity of the structures enormously.

**Figure 1:**
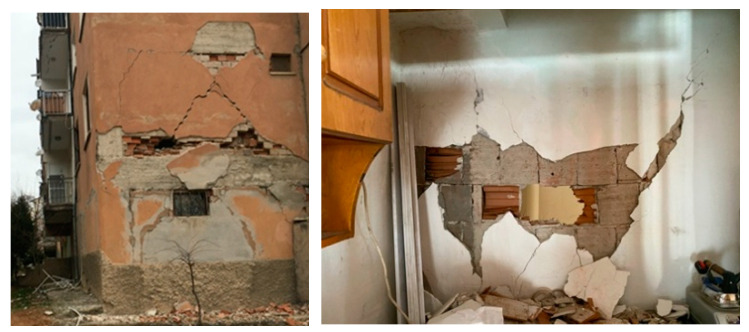
In-plane and out-of-plane infill walls damages (Elazig Earthquake, 2020).

In general, a flexible and ductile structure can sustain severe earthquakes exhibiting large displacements. Meanwhile, the brittle components of the structural systems, such as masonry infill walls, may suffer from substantial damages due to excessive drifts [1,40]. Such damages due to in-plane and out-of-plane actions observed after previous earthquakes are shown in Figure 1. To avoid in-plane and out-of-plane infill wall damages, seismic design documents limit the inter-storey drifts based on the interface conditions between the infill and surrounding structural frame, and require to check the stability of infills and the safety of their connections to the frames [41,42]. Flexible frames are unable to carry high loads at low displacements and this can cause the infill to damage already at moderate seismic intensity. In case of aftershocks, the damaged infills can fail out-of-plane. In case of infill damage, polyurethane (PU) can be used for bonding of various composite fibres to the weak masonry substrate to form Fibre Reinforced Polyurethanes (FRPU) as well as for repair of damaged RC frames. The PU can be used in emergency situations, as it cures within hours and is easy to apply. Moreover, PU may be used to create flexible joint PUFJs) between frames and infills to form an innovative protection in the frame-infill interface. Several previous studies show that the response of structural systems can be improved by using PUFJ or/and FRPU [38,39,43,44].

The present study reports the experimental results of the “infills and masonry structures protected by deformable polyurethanes in seismic areas” (INMASPOL) project within the SERA, Horizon 2020 framework. INMASPOL investigates the efficiency of the innovative PUFJ protection and of the FRPU as emergency repair or strengthening. Both methods are applied on full-scale infilled RC structure tested on shake table under simulated seismic excitations. The seismic tests validated the improved in-plane and out-of-plane infill performance when modified or repaired with PUFJ and FRPU systems (technology protected by patent application to EU Patent Office). This paper highlights the key role of the flexible polymers used in both PUFJ and FRPU.

## 2. Experimental Setup

**Figure 2:**
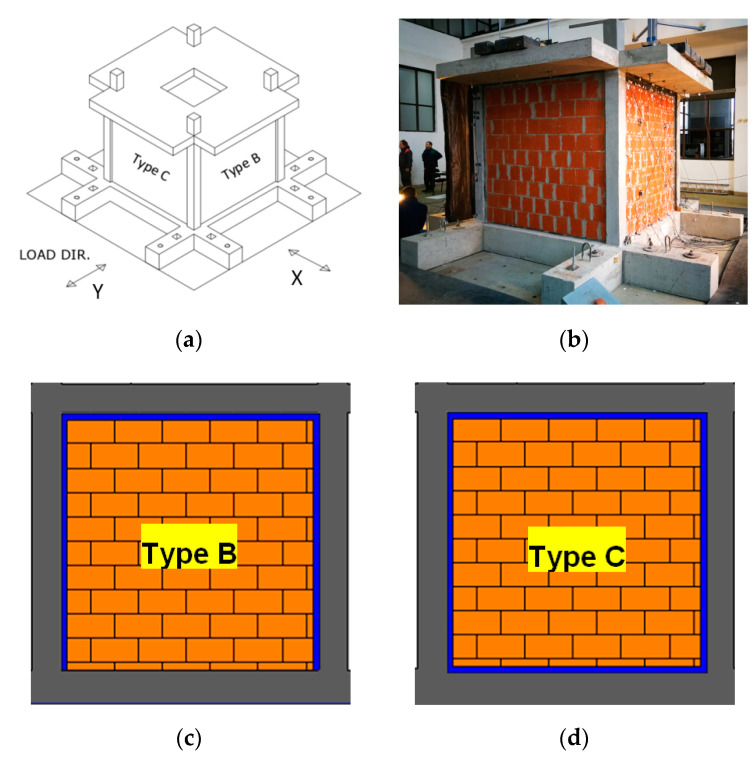
Experimental model: 3D drawing, (**a**) specimen view, (**b**); schemes of infill: type B, (**c**) and type C, (**d**).

The tested specimen was a fully symmetrical 3D frame of one story with four RC columns, four beams, a slab and four infill masonry walls, all designed according to current Eurocodes 2 and 8. The real scale building had plane dimensions of 3.8 m × 3.8 m and height of 3.3 m (foundation and column extensions included, Figure 2a,b).

### 2.1. Materials

**Figure 3:**
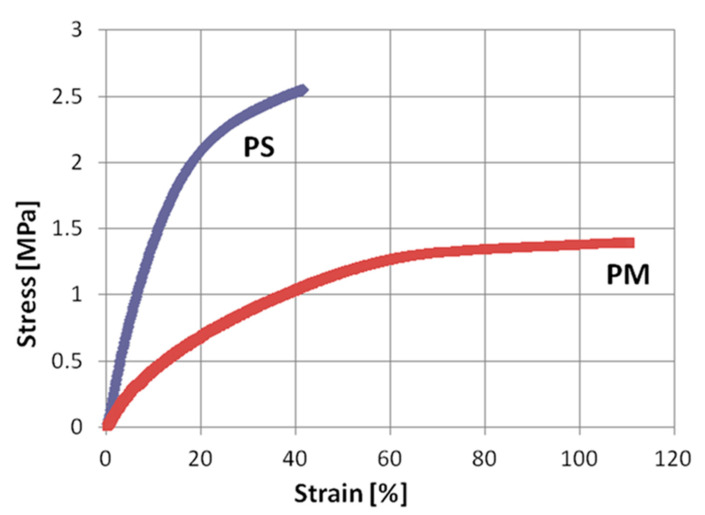
Stress–strain curves of polymers Sika PM and Sika PS.

Concrete used in construction was produced in a ready-mix concrete plant. The compressive strength of concrete cubes with 15 cm side at 28 days was 34.1 MPa for the foundation, 27.1 MPa for the columns and 34.2 MPa for the slab. Average mass density of concrete was 2380 kg/m^3^. Steel for reinforcement was grade B 500B with characteristic yield strength 500 MPa. Blocks for infill walls were hollow clay units KEBE OrthoBlocks K100 (Kilkis, Greece) with dimensions 100/240/250 mm and weight of about 100 kg/m^2^ of a wall with vertical holes. Mortar for construction was OrthoBlocks mounting mortar in the form of a dry ready-mix, with nominal strength class M10. The mortar was laid in thin layers of 3 mm thickness. Both, head and bed joints, were filled with the same mortar thickness. Polymer for PUFJ was of type Sika PM. The elastic modulus, strength and ultimate elongation of the polymer were 4 MPa, 1.4 MPa and 110%, respectively in a uniaxial tensile test [45]. Polymer for FRPU was of type Sika PS. The elastic modulus, strength and ultimate elongation of the polymer were 16 MPa, 2.5 MPa and 40%, respectively [45]. Stress–strain curves of both polymers are compared in Figure 3. GFRP (Glass Fiber Reinforced Polymer) mesh of type Sika Wrap 350G Grid, made of glass fibre reinforced polymer mesh, was used for the repair of the walls. The mesh of a real weight 360 g/m^2^ had the elastic modulus, strength and ultimate elongation of the polymer were 80,000 MPa, 2600 MPa and 4%, respectively in a tensile test (producer data). Both resins used in the joints’ construction or to impregnate and bond the GFRP mesh, were ultra-flexible as their modulus of elasticity was around 4–16 MPa. Further, they were ultra-high deformable as their ultimate tensile elongation ranged from 110% to 40% respectively. These mechanical characteristics ensured their advanced deformation compatibility with adjacent brittle materials even after these materials entered the damage accumulation stage.

### 2.2. The Structure

The one storey RC frame specimen with infills had a 1:1 scale and was designed according to shaking table capacity. The floor plan dimensions of the frame were 2.7 m × 2.7 m, and the 20 cm × 20 cm columns had a height of 2.5 m (to the top of the slab). The height of infills in the building was 2.3 m (Figure 2a). The structure was designed according to Eurocodes 2 and 8 requirements. The columns were reinforced with 8 ø 10 longitudinal rebars (three bars, symmetrically placed at each column side) and 2 ø 8/50 mm stirrups (square and diamond stirrups to restrain effectively all eight bars of the section). The beam was hidden inside the slab, and reinforced by the same amount of longitudinal reinforcement (three bars, symmetrically placed at each beam side), and by ø 8/50 mm stirrups (square stirrups to restrain effectively only the four corner bars). The clear concrete cover to the stirrup was 42 mm for the columns and the beams (effective depth d = 55 mm to the longitudinal bars). On top of the columns, an RC slab with a thickness of 20 cm was constructed. The slab was extended beyond the frame beams to serve as additional mass and to attach the additional masses in the form of steel ingots. In the middle of the slab there was a hole for access to the inside of the model (see Figure 2a). The slab was reinforced with welded meshes of Q503 at the top and at the bottom. There was additional reinforcement at the perimeter edges of the slab, and at the edges of the hole. The model structure was constructed on a special foundation, with holes for attaching it to the shake table and hooks for lifting and manipulating the structure (Figure 2b).

The infills too were designed according to the shaking table capacity and in relation to the RC frame strength and stiffness. They were made of hollow clay blocks (OrthoBlock type) with thickness of 10 cm, typical for internal infills. Two parallel walls were of type B, and the other two walls were of type C. Type B infill (Figure 2c) was constructed directly on the foundations, while there was a 2 cm thick in-situ produced PUFJ (by injection of polyurethane Sika PM) between the infill and the RC columns, and between the infill and the RC slab. The PUFJ soft joint was thus on three edges (left, right and top) and had a chemical bond to RC and infill surfaces due to in-situ application. Curing PU was attached to the dust-free RC surface covered with special PU primer, which ensured compatible interface between polymeric and concrete materials. Adhesiveness of Sika PM to brick substrates was determined by a pull-off test in [18], where value of 0.63 MPa was obtained and adhesive brick/polymer failure mode was observed. Type C infill (Figure 2d) was constructed on a prefabricated 2 cm thick PUFJ joint (made of polyurethane Sika PM) bonded to the RC foundation beam and the same type of joint was at the sides and top of the infill. The prefabricated PUFJ was thus bonded on all four edges (top, bottom, left and right) of the frame using chemical bond to RC surfaces (made of polyurethane Sika PS) but through the mortar to infill surfaces. Adhesiveness of Sika PS to brick substrates was determined by a pull-off test in [18], where value of 1.48 MPa was obtained and adhesive brick/polymer failure mode was observed. The same OrthoBlocks mounting mortar (with nominal strength class M10) was laid in thin layers of 3 mm thickness, as in head and bed joints. Thus, the building received two different innovative resilient protection schemes using Polyurethane Flexible Joints (PUFJs) made of polyurethane resin (PU) at the frame-infill interface. The Orthoblocks are similarly vital component of the protection scheme as they are bearing-masonry-oriented and thus their holes are at the vertical direction of the infill. This feature suggests that the compressive strength of the infill is higher at the vertical direction (contributing to the anti-collapse capacity of the RC frame) than at the horizontal one (contrary to the typical infills constructed with brick layered with their holes at the horizontal direction). Only one scheme per pair of infills in the same direction was designed: the two infills in the Y direction received in-situ injected PUFJ (type B, Figure 2a,c) and the two infills in the X direction received prefabricated PUFJ (type C, Figure 2a,c). At first, the structure was tested at Y direction (type B infills were subjected under in plane testing and type C infills under out of plane testing). Afterwards, the structure was rotated by 90° and the type C infills were subjected under in plane testing while type B infills out of plane. The approach of symmetric interventions in each pair of the infills in the same direction was followed to avoid undesirable non-symmetrical resistance of the paired frames. Therefore, it is considered that each infill of the same pair develops the same base shear force during in plane loading. On top of the structure, there were 18 steel ingots uniformly distributed with a total mass of 7200 kg. The structure on the shake table is shown in Figure 2b.

### 2.3. Testing Facility and Equipment

The shake table tests were performed in the laboratory for dynamic testing in the Institute of Earthquake Engineering and Engineering Seismology in Skopje (IZIIS), Republic of North Macedonia. Operational since the year 1980, the one direction excited (Y-direction—see Figure 2a) shake table was 5.0 m by 5.0 m pre-stressed concrete waffle slab weighing 33,000 kg with payload up to 40,000 kg. In total, five degrees of freedom were provided by 2 lateral and 4 vertical MTS hydraulic pistons, controlled by MTS Digital Controller 469D. National instruments PXI modular system was used for data acquisition for the three different types of transducers: 23 accelerometers, 10 linear variable differential transformers and two linear potentiometers. 

### 2.4. Instrumentation

**Figure 4:**
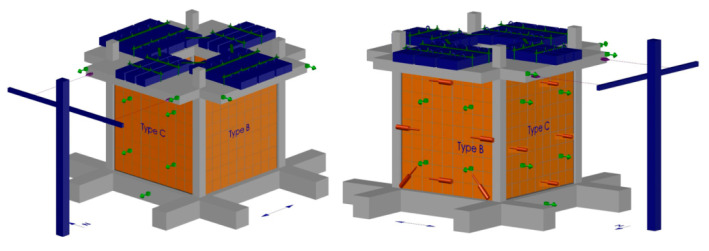
Scheme of instrumentation.

The instrumentation consisted of 23 accelerometers located on points indicated by green arrows in Figure 4. The accelerometers were used to measure in- and out-of-plane accelerations of the infills, and the accelerations of the top slab and of the foundation. A total of eight LVDTs (Linear Variable Differential Transformers) were used to measure relative displacements between the infills and the RC frame structure on the in-plane and out-of-plane loaded infills. Then, two additional LVDTs were used to measure diagonal deformation of the infill. In total, there were 10 LVDTs, which are indicated by red markers in Figure 3. Two linear transducers were used to measure the top drift of the structure (blue markers) and two attached to the support, to measure relative displacements of the slab). In addition, optical system was used to measure displacement fields on both in-plane loaded walls, to measure absolute displacements of the frames and the infills. 

### 2.5. Applied Testing Methodology

For the purpose of the experimental investigations, two types of tests were performed, i.e., dynamic shake table tests and tests for determination of dynamic characteristics. The dynamic shake table tests comprised of gradually increasing input intensity level from 3% to 77% of the adopted earthquake Kefallonia E-W component, simulating a critical excitation, causing out-of-plane failure of the most vulnerable wall infills (see Section 2.6). The maximum applied load level was dependent on the induced damage of the RC frame and infill walls as well as on the limit state of the shake table (considering the weight of the specimen and the additional load).

### 2.6. Seismic Loading

**Figure 5:**
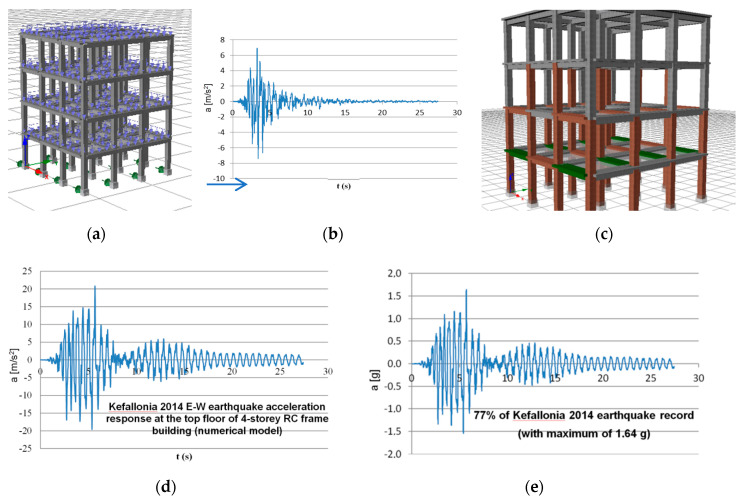
Typical four-storey building (**a**), suffering Kefallonia 2014 E-W (Chavriata) earthquake, (**b**) and damage levels, (**c**). Kefallonia 2014 E-W (Chavriata) earthquake acceleration response at the top floor of 4-storey building (reference signal), (**d**) and scaled acceleration loading for 77% of the reference signal (KEF), (**e**).

The test structure represents in- and out-of plane loaded walls at the top of 4-storey RC building. In order to generate the excitation for the seismic table, a numerical inelastic model of a typical 4-storey RC frame building (Figure 5a) was subjected to the actual Kefallonia 2014 (Chavriata) earthquake record in E-W direction (ground acceleration with a_max_ = 0.75 g in Figure 5b). This earthquake excitation was recorded at the Chavriata station in the near-field, within the rupture zone and with 7 km distance from the earthquake epicenter. It presented extremely high values of spectral accelerations, around 2.6 g at 0.25 s and at 0.4 s (far higher that the corresponding provisions by Eurocode or Greek Codes). Given the potential of using only one record to subject our structure to successive excitations of increasing accelerations (equivalent to dynamic pushover testing) up to its damage, this record was considered characteristic for catastrophic near-field earthquakes. The damage level of the analysed building is shown in Figure 5c. Brown colour denotes internal steel yielding and green colour denotes crushing of unconfined (cover concrete) after the excitation which resulted in a maximum structure top displacement of 14 cm and relative drift of 0.01 (1%). Then, the acceleration response (with a_max_ = 2.12 g) at the top floor of the structure (Figure 5d) was decided to be the most detrimental for out-of-plane failure of the assumed infills in a similar wall infilled structure. This top floor excitation was slightly modified to conform to the shake table capacity and get a better reproduction of the input signal. The Kefallonia E-W component dominant frequency range was 2.5–4.0 Hz (after Fast Fourier Transform analysis). The applied maximum acceleration history, scaled to 77% of the Kefallonia 2014-based earthquake record (77% KEF-1—with a_max_ = 1.63 g)) is the input signal for the tests and is shown in Figure 5e.

### 2.7. Experimental Program

**Figure 6:**
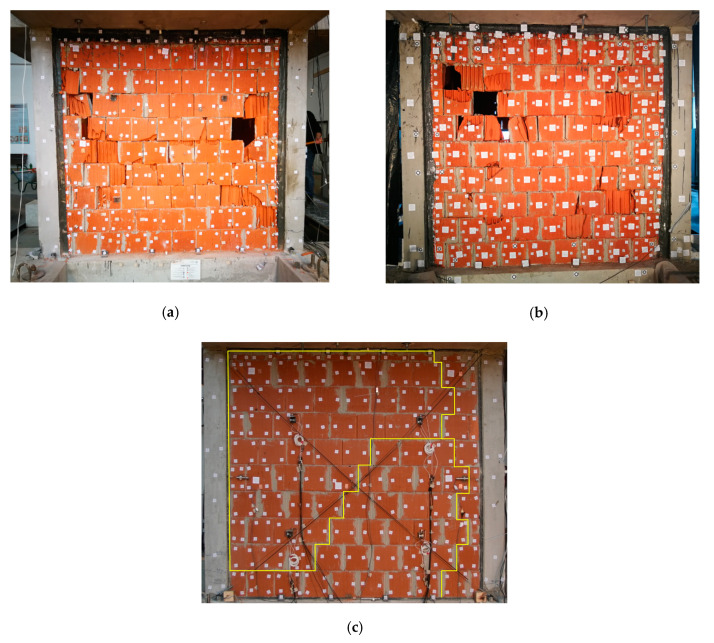
Infill type B wall with significant damages after PHASE 1, north side (**a**), and south side (**b**), Fissures pattern in the infill C after PHASE 1 (**c**).

The structure was always tested by a uniaxial dynamic excitation and after selected earthquake runs, the eigen-frequencies were measured by loading the structure with white-noise (random) excitation. The original position of the model was such that both type B walls were loaded in-plane, and both type C walls were loaded out-of-plane (Figure 2b and Figure 4). In this position (PHASE 1), the dynamic load was gradually increased until there were significant in-plane damages in infills B (Figure 6a,b), which occurred during 77% KEF–1—see Table 1.

There were also damages in concrete columns (at the top and at the bottom). Fissures in head and bed mortar joints of infills C were also detected at this stage, caused by shear forces during out-of-plane excitation with 77% KEF-1. The fissures pattern is presented in Figure 6c. In PHASE 2, the type B walls were repaired by a single layer of glass FRP mesh on both sides, bonded to the damaged wall using flexible adhesive of type Sika PS (FRPU repair), without any special treatment of the infill face and no crack repair. The mesh was applied in two diagonal strips and at all the edges of the infills, without direct connection to the RC frame (the repaired infills B were connected with the RC frame only through the previously injected PUFJs). It should be noted here that there was no plaster on the walls during all phases of the experimental campaign.

**Figure 7:**
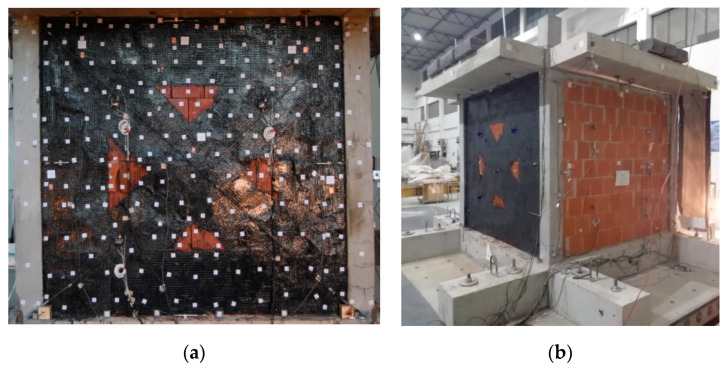
Strengthened type B walls, front view, (**a**) and perspective view, (**b**).

The width of all applied glass FRPU strips was 50 cm. The layout of the FRPU intervention is shown in Figure 7. The FRPU was applied on both sides of the damaged B infills (B_FRPU), without additional connections between inner and outer sides. The aim of this strengthening was to protect the damaged infills against collapse during further in-plane tests of the B_FRPU in PHASE 2 and during out-of-plane excitation of the B_FRPU in PHASE 3, after rotation of the building by 90°. This type of emergency repair/retrofit of damaged infill can be applied in real applications, as a protection against out-of-plane collapse against aftershocks. Once quick emergency repairing was applied, the model was again loaded only 10 h after FRPU application by gradually increasing seismic loading (see Table 1 for details—PHASE 2).

**Figure 8:**
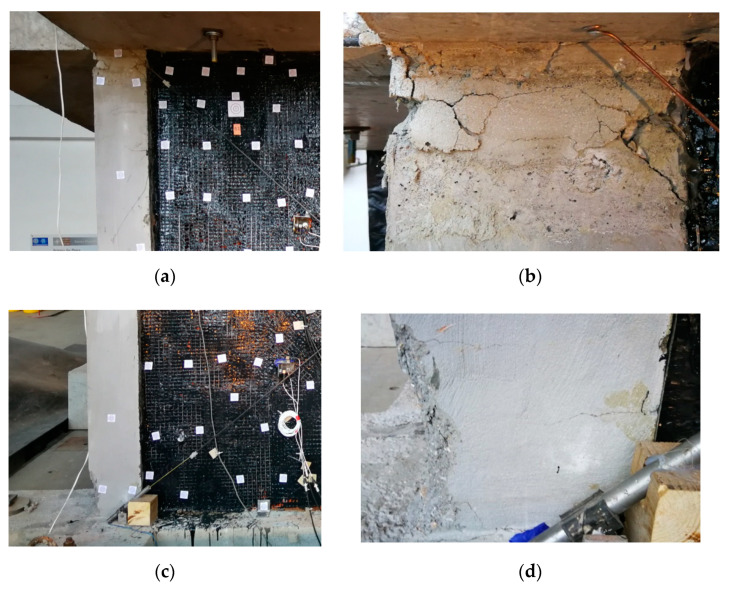
Cracked concrete at the RC column top region, (**a**) (and focus, (**b**)) and column bottom region, (**c**) (and focus, (**d**)).

First, out-of-plane tests with harmonic resonance frequencies (16 Hz and 32 Hz) of the infills C were additionally carried out up to 40% KEF, after PHASE 1 but before PHASE 2, to check the out-of-plane resistance of the slightly damaged infills C (without any strengthening or repair). No significant damages to the infills C were observed (only widening of the existing fissures in mortar (Figure 6c). Next, gradually increasing seismic loading of PHASE 2 (see Table 1 for details) was applied. It should be noted that the building was not rotated during the second stage of excitations. Therefore, the walls that were subjected to in-plane actions in the first stage, were again subjected to in-plane actions in the second stage. At 18% KEF-2 intensity, the testing was stopped in order to avoid uncontrolled damage to the RC structure (crushing of concrete at the top and the bottom of columns were observed—see Figure 8) that could jeopardise the third phase of the tests. Fissures of the infills C (Figure 6c) did not practically change during PHASE 2. The infills B strengthened by FRPU (Figure 8a,b) did not practically change their form and no additional damages were observed after PHASE 2.

In the next step, the entire structure was rotated by 90° on the shake table. In PHASE 3, the strengthened type B walls were loaded in the out-of-plane direction, and the type C walls (with the crack pattern presented in Figure 6c) were now loaded in the in-plane direction. The intensity was gradually increased until 16% KEF-3 intensity, when observable damages to infills C were noticed (by visual inspection and by changes in the eigen-frequency of the structure). The sequence of intensities is presented in Table 1. It should be noted that the building was rotated before the third stage of excitations. Therefore, the walls subjected to in-plane actions in the first and second stages (infills B), were subjected to out-of-plane actions in the third stage (in PHASE 3).

At 16% KEF-3 intensity, the testing on the rotated structure was stopped in order to avoid further damage to the RC structure and the masonry infills C. Before stopping the test, an increase of structural softening and visible temporary openings in location of fissures in head and bed mortar joints (shown in Figure 6c) were observed. The fissures on infills C did not change the previous form after stopping of the tests of PHASE 3. Another reason of stopping the test was to protect the weakened infills C to be able to test their performance under in-plane action after a later FRPU strengthening intervention.

## 3. Test Results

Shake table tests were carried out for various earthquake excitation levels (% KEF), described in Table 1. Direction of the shake table excitation was marked as Y and the perpendicular direction as X (Figure 2a). Analysis of results was carried out first for horizontal relative displacements of the slab related to the building foundation (Figure 4).

### 3.1. Infills of Type B Tested In-Plane

**Figure 9:**
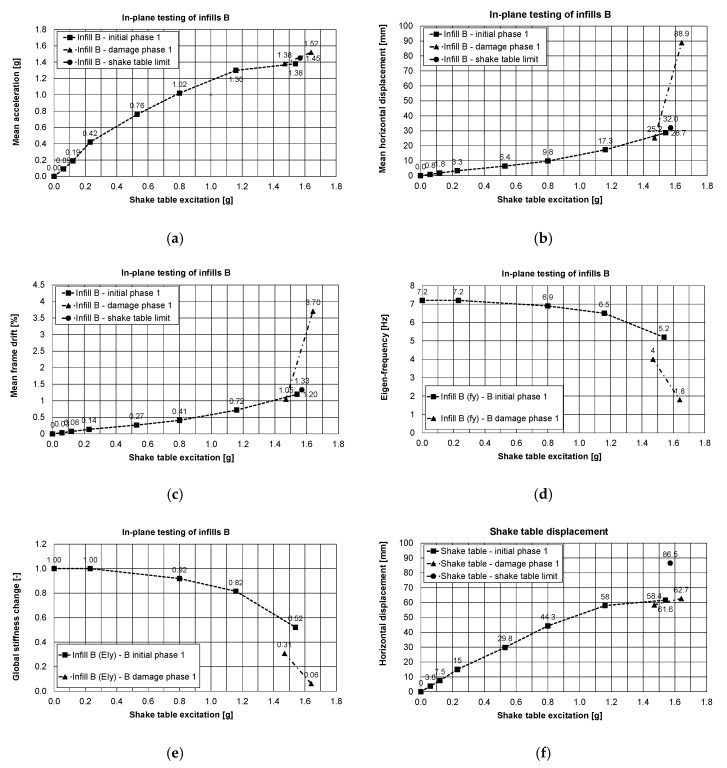
In-plane testing results of B infills, PHASE 1: mean acceleration of the slab, (**a**), mean displacements of the slab, (**b**), mean drift, (**c**), eigen-frequency, (**d**), global stiffness changes in direction of in-plane of B, (**e**) and shake table displacement, (**f**).

**Figure 10:**
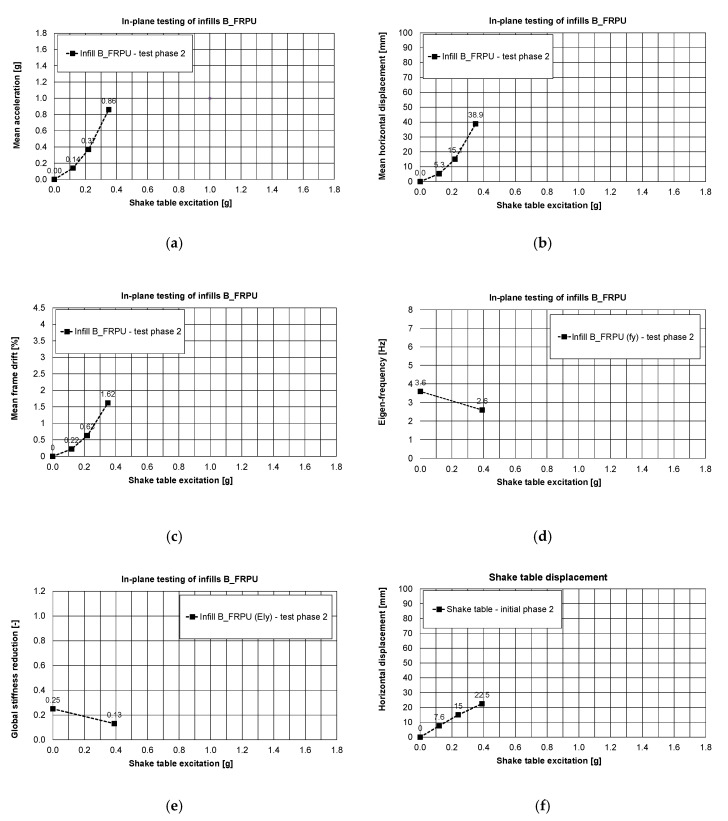
In-plane testing results of infills B_FRPU, PHASE 2: mean acceleration of the slab, (**a**), mean displacements of the slab, (**b**), mean drift, (**c**), eigen-frequency, (**d**), global stiffness changes in direction of in-plane of B-FRPU, (**e**) and shake table displacement, (**f**).

**Figure 11:**
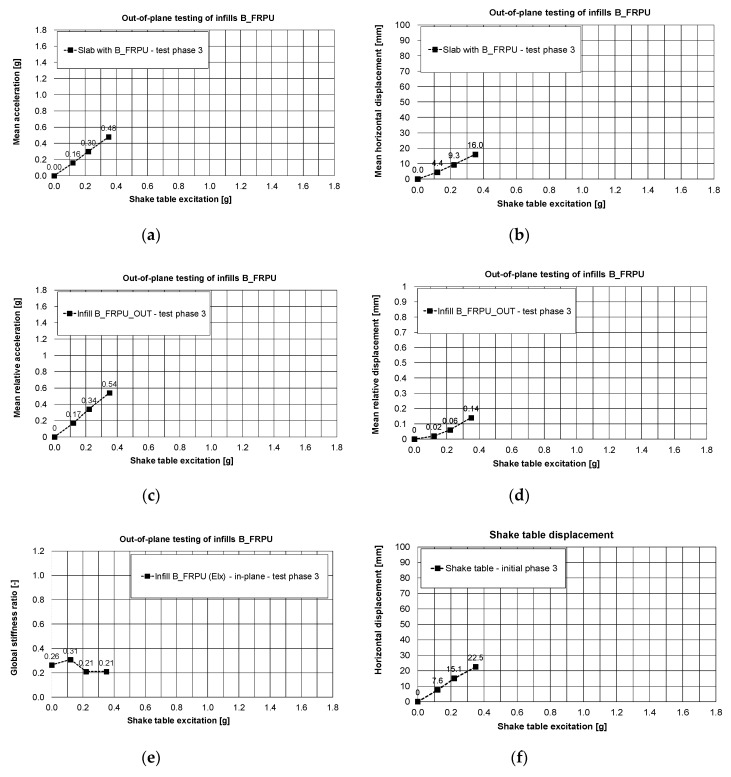
Out-of-plane testing results of infills B_FRPU, PHASE 3: mean slab acceleration, (**a**), mean slab displacements, (**b**), mean relative acceleration of B-FRPU out-of-plane, (**c**), mean relative displacement of B-FRPU out-of-plane, (**d**), global stiffness changes in direction of in-plane B-FRPU, (**e**) shake table displacement, (**f**).

Referring to Table 1, Figure 9 and Figure 10 show the in-plane testing results of the infills type B obtained for PHASES 1, 2 and Figure 11 shows the out-of-plane ones obtained for PHASE 3. Figure 9 shows the mean values (mean is the average of two sensors measuring acceleration along the same direction) of: acceleration of the slab (Figure 9a), displacements of the slab (Figure 9b) and drift (Figure 9c) induced by the shake table displacements illustrated in Figure 9f. The eigen-frequency and the stiffness changing of the building are illustrated in Figure 9d,e. A similar pattern of sub-figures is applied for Figure 10 and Figure 11, obtained in PHASE 2 and in PHASE 3, respectively.

In PHASE 1, the infills B were tested in-plane in an initial phase up to 72% KEF-1 intensity (see Table 1), where damage of the infills B was hardly observed. When the structure was again loaded up to 74% KEF-1 intensity, the capacity of the table was exhausted by movement of the specimen on the shake table surface (causing activation of the table security overloading sensor and resulting in sudden stop of the test—shake table limit not registered by the acquisition system in the safe mode, only the last registered data are presented with black dot in Figure 9a–c). As the result, additional damages in the building occurred due to sudden stop of the shake table and activation of additional inertial forces, but without visible degradation of the specimen (up to this point, results are presented by main curves in Figure 9). After checking and correction in the shake table system, the infills B were tested further in the damage phase with 69% KEF-1 and 77% KEF-1 intensity (Table 1, results are presented by additional curves in Figure 9). Formation of significant damages (Figure 6) were observed during the final excitation. The observed crack pattern was different from the classical cross shape (><) going through corners. The corner zones were protected against cracking by the PUFJs and thus damages were localised in the middle of the infill height, forming the horizontally extended cross shape (>--<).

Therefore, in PHASE 1, the tested building withstood (without serious damages to the B infills—initial phase) the maximum shake table excitation up to 1.57 g base acceleration and 86.5 mm of base displacement (Figure 9f). The corresponding recorded maximum of mean values were: a slab acceleration equal to 1.45g (Figure 9a) with slab relative displacement equal to 32 mm. The corresponding maximum horizontal (Y-direction—see Figure 2a) drift was 1.33% for the RC frame, using the height of the columns up to the centre of the joint with the beams 240 mm deep. During repetition of excitation (1.47–1.64 g base acceleration and 58.4–62.7 mm base displacement—Figure 9f), the initiation of significant damages occurred (damage phase) under 2.5% mean drift, corresponding to the relative slab displacement equal to 60 mm.

The largest mean values obtained in the damage phase for 1.64g base acceleration (after occurrence of significant damages to the B infills) were: mean acceleration equal to 1.52 g (Figure 9a) and the mean displacement equal to 88.9 mm (relative bottom to top displacement corresponding to drift of the columns equal to 3.7%). The PUFJs protected the infills B against total disruption and collapse (Figure 6), even when the drift exceeded significantly the value of 0.5% for non-structural elements or even the 2% value.

The behaviour of the structure was almost linear up to 38% KEF-1 intensity (0.80 g). Softening started once plastic hinges were developed at the top and at the bottom of the columns and cracking occurred in the head and bed mortar joints at 55% KEF-1 intensity (1.16 g), which further developed when excitation continued up to 77% KEF-1 intensity (1.64 g). 

**Figure 12:**
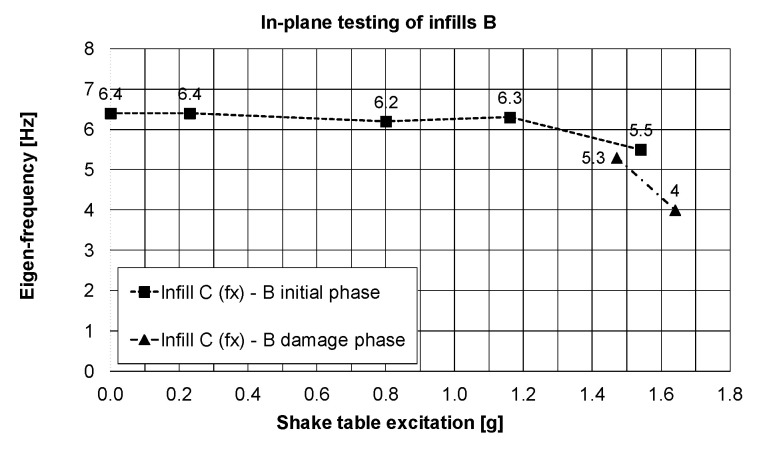
Changes of the main eigen-frequency of the building in the X-direction (in-plane of the infills C) when the infills B were tested in-plane (Y direction) and the infills C out-of-plane, PHASE 1 and 2.

Observation related to the drift changes are confirmed by the reduction of the main eigen-frequency of the building in the Y-direction from 7.2 Hz to 1.8 Hz, obtained from the white noise tests. Significant damage to infills B appeared in the 77% KEF-1 intensity. These correspond to reduction of the eigen-frequency below 4 Hz and interfering in resonance with the dominant excitation frequency of the earthquake signal (2.5–4.0 Hz). Changes of the global stiffness of the building are presented in Figure 12, in the form of a stiffness ratio, defined by the equation:

(EI_current_)/(EI_initial_) = (fy_current_)^2^/(fy_initial_)^2^

where EI_current_ = current stiffness; EI_initial_ = initial stiffness; fy_current_ = current eigen-frequency; fy_initial_ = initial eigen-frequency. Reduction of the global stiffness up to 31% of the initial stiffness before the tests was caused mainly by formation of plastic hinges in the RC columns and loss of cohesion in head and bed mortar joints. Therefore, the global stiffness of the building (after these serious damages) dropped significantly to 6% of the initial stiffness before the tests. This occurred within 77% KEF-1 intensity excitation, but the structure did not collapse, and no significant permanent deflection was observed. Moreover, the blocks were kept by the PUFJs on the position with only single blocks rushing out. It was possible to repair/strengthen the infills B (in such condition) using glass FRPU system (Figure 7). After strengthening the infills B, the structure revealed increased eigen-frequency in the Y direction up to 3.6 Hz (recovery of stiffness to the level of 25% of the initial stiffness before the tests). 

In PHASE 2, the tested building (after FRPU repair of the damaged B infills) withstood (without serious damages to the B_FRPU walls) a shake table excitation with 0.39 g base acceleration and 22.5 mm of base displacement (Figure 10f). The corresponding maximum of mean values were: a slab acceleration equal to 0.89 g (Figure 10a) with slab displacement equal to 38.9 mm—Figure 10b (drift of 1.62%—Figure 10c). The FRPU repair effectively protected the damaged B infills against total disruption and collapse—no additional damages were observed in B_FRPU walls (Figure 7). Before the PHASE 2 tests and after FRPU repair, the building global stiffness increased significantly—over four times—from 6% to 25% of the initial one. It corresponded to the eigen-frequency shift from 1.8 Hz to 3.6 Hz.

During PHASE 2, the building with B_FRPU walls was excited in the range of its resonance frequency band (2.5–4.0 Hz)—see Figure 10d. After in-plane testing of the strengthened infills B up to 18% KEF-2 (0.39 g) in PHASE 2, no additional damages were observed. At the end of this phase, the global stiffness of the building dropped to about half, from 25% to 13% of the initial one. It corresponds to an eigen-frequency shift from 3.6 Hz to 2.6 Hz. No additional damages were observed in the B_FRPU walls after the completion of PHASE 2 (Figure 7).

Before PHASE 3, the tested building was rotated by 90°, thus the B_FRPU infills would mainly be subjected to out-of plane actions in PHASE 3. Oppositely, in PHASE 3, the infills C were subject to mainly in-plane actions. The B_FRPU infills withstood, without additional damages, out-of-plane earthquake excitations with 0.35 g base acceleration and 22.5 mm of base displacement (Figure 11f).

The corresponding mean values were: a slab acceleration equal to 0.48 g (Figure 11a) and a relative slab displacement equal to 16.0 mm—Figure 11b (mean drift of the structure was equal to 0.67%—measured in-plane of the infills C). The FRPU repair effectively protected the damaged B infills against out-of-plane fall-out or total disruption and no additional damages were observed in B_FRPU walls. The relative mean acceleration and relative mean displacement between the infill B and the RC frame (corresponding to 0.35g base acceleration and 22.5 mm of base displacement), measured for out-of-plane movement of the B_FRPU infill were equal to 0.54 g and 0.14 mm, respectively (Figure 11c,d). Before the building rotation, its global stiffness in direction of in-plane B_FRPU (Y direction) was 13% of the initial one. After rotation, it increased to 26% in the same plane (X direction).

During PHASE 3, the building with B_FRPU infills tested out-of-plane was once more excited in the range of its resonance frequency band (2.5–4.0 Hz)—the building eigen-frequencies in both directions varied between 3.3 Hz and 4.0 Hz. As a result, the building global stiffness in direction of in-plane B_FRPU dropped from 26% to 21% of the initial one. It corresponds to an eigen-frequency shift from 3.7 Hz to 3.3 Hz. The presented results related to the infills B indicate that PUFJ systems effectively protect RC frames and infill walls during strong earthquakes. Similarly, glass FRPU systems efficiently protected the totally damaged (disrupted) infills B against falling out during moderate earthquakes (e.g., aftershocks).

### 3.2. Infills of Type C Tested In-Plane

In PHASE 1 (the infills B tested in-plane), the infills C were tested out-of-plane in the initial phase up to 77% KEF-1 intensity and then in the damage phase with 69% KEF-1 and 74% KEF-1 intensity (Table 1). Practically no serious damages to the infills C (only fissures in mortar—Figure 6c) were observed at this stage after very high acceleration out-of-plane excitations and also after the harmonic resonance tests with 16 Hz and 32 Hz frequencies (for out-of-plane excitation of the infills C). The infill was protected by PUFJs against detachment from the RC frame, which allowed the infill to move out-of-plane like a stiff slab with the eigen-frequency of 16 Hz and like a bending slab with the eigen-frequency of 32 Hz. The crack pattern visible in Figure 7 corresponds to damage of a simply supported slab. Degradation of RC columns resulted also in changes of eigen-frequencies in the perpendicular X-direction, from 6.4 Hz to 4.0 Hz (Figure 12), close to the resonance frequency range of the KEF earthquake excitation. Excitation of the structure in PHASE 2 did not change the eigen-frequency of the building in X-direction (left 4.0 Hz).

**Figure 13:**
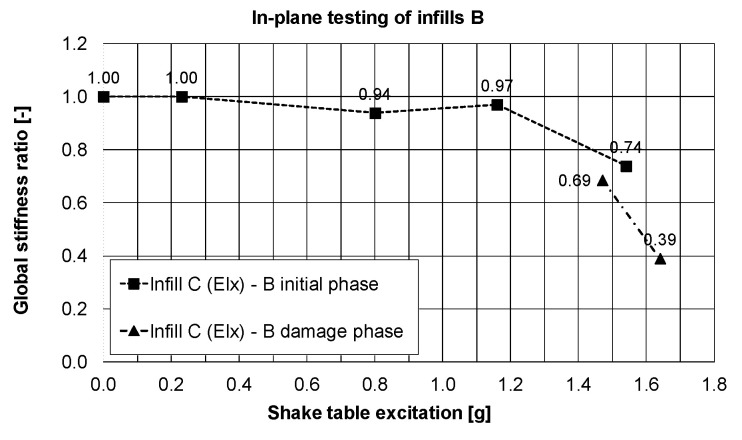
Changes of the global stiffness of the building in the X-direction direction (in-plane of the infills C) when the infills B were tested in-plane (Y direction) and the infills C out-of-plane, PHASE 1 and 2.

Reduction of stiffness in the X-direction of excitation up to 39% after PHASES 1 and 2 was calculated from Figure 13. Influence of the building rotation and of action of additional shear forces were observed in change of eigen-frequencies and stiffness. The building had variable frequencies (and stiffness): in-plane of the infills C—decreased from 4.0 Hz (stiffness of 39%) to 3.5 Hz (stiffness of 30%), whereas in-plane of the infills B—increased from 2.6 Hz (stiffness of 13%) to 3.7 Hz (stiffness of 26%).

**Figure 14:**
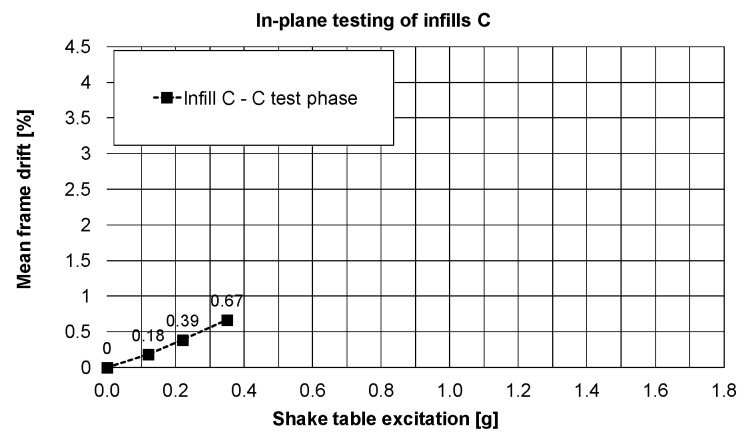
Changes of calculated drift of the slab, PHASE 3.

After rotation of the building, the infills C presented slightly non-linear behaviour during in-plane tests in PHASE 3. The drift changes calculated based on the maximum relative horizontal displacement of the slab are presented in Figure 14 for different excitation intensities up to 16% KEF-3.

**Figure 15:**
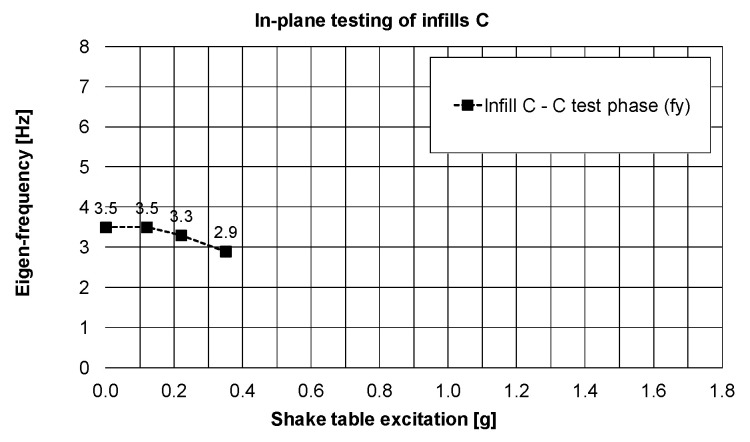
Changes of the main eigen-frequency of the building in the Y-direction, PHASE 3.

**Figure 16:**
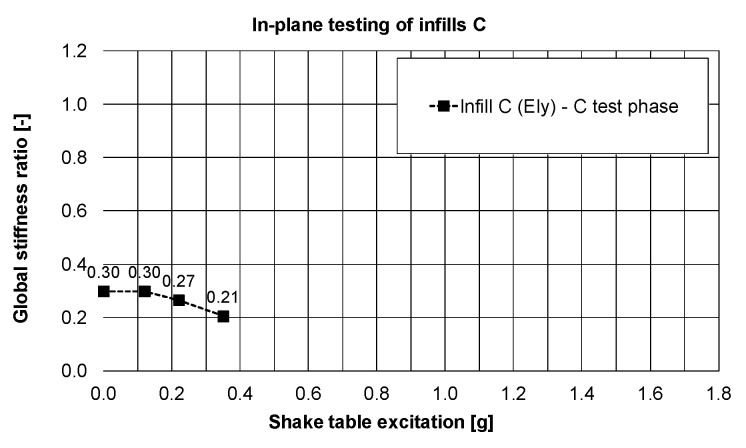
Changes of the global stiffness of the building in the Y-direction, PHASE 3.

Observation related to the drift changes were confirmed by small reduction of the eigen-frequency—Figure 15 (and stiffness—Figure 16) in the Y-direction: from 3.5 Hz (stiffness of 30%) to 2.9 Hz (stiffness of 21%), even if the structure worked in the resonance frequency range of the KEF earthquake excitation (2.5–4.0 Hz).

It is worth noticing that the initial building stiffness in the direction of the infills C plane (related to 6.4 Hz frequency—Figure 12) was 21% lower than the initial building stiffness in the direction of the infills B plane (related to 7.2 Hz frequency—Figure 9d). This was caused by the influence of the PUFJ working at the bottom of the infills C.

## 4. Conclusions

The described shake table tests on a full-scale RC frame with infills showed that infills significantly influence the seismic response of the structure. Under suitably designed seismic excitations, the tests validated the superior in-plane and out-of-plane performance of infills protected by using innovative polyurethane solutions. The detrimental effects of strong earthquakes on the stiffness and the eigen-frequencies of the structure—both denoting damage accumulation—are better controlled in case the hollow clay blocks (orthoblock) infill-RC frame boundaries are protected with PUFJ joints. The use of these PUFJs at the infill-RC frame interface enabled a quick repair of the infill even after very high inter-storey drift of the structure. It was observed that due to the flexibility of the 2 cm thick polymer joint, the interaction between the special orthoblock infill and the RC frame can be manipulated so as to achieve the delay of significant infill damages at very high RC frame inter-storey drifts. The present tests suggest that first brick disintegration (that may cause injuries) occurs at 2.5% drift while avoiding undesirable effects on the RC columns, caused by the infills. Further, it is validated, during the same run of excitation, that the PUFJ protection enabled the infill to be repaired, via innovative FRPU, even after the frame has been subjected to very high inter-storey drifts up to 3.7%. This drift level is higher than the one corresponding to repairable damages for ordinary infilled RC frames. The applied glass FRPU system efficiently protected the damaged orthoblock infills against collapse, under in plane or under out-of-plane excitation, while it restored large part of their in-plane stiffness as well. 

The tests present a direct comparison between the cases with repairing injected PUFJs on three sides (left, top, and right) and with pre-installed prefabricated PUFJs on all four sides for low excitation levels. Both PUFJ systems protected efficiently the orthoblock brick infills against out-of-plane failure for very high inter-storey drifts and accelerations.

It should be noted that both resins used in the joints’ construction or to impregnate and bond the glass FRP mesh, are ultra-flexible as their modulus of elasticity is around 4-16 MPa. Further, they are ultra-high deformable as their ultimate tensile elongation ranges from 110% to 40% respectively. These mechanical characteristics ensure their advanced deformation compatibility with adjacent brittle materials even after these materials have entered damage accumulation stage.

The flexible FRPU system achieved excellent bonding with the orthoblock infill substrate throughout the successive dynamic tests (no bonding on the concrete faces was necessary). It ensured safety against infill collapse, despite damage accumulation within the infill and the RC members. Finally, it showed no interfacial bond degradation. This is one of the main properties of PU in comparison to epoxy resin or to other stiff resins: the better stress distributions allow it to not damage or to delay the damages, both on the fibres and on the substrate.

## Figures and Tables

**Table 1:** Test phases of the infills type B and list of earthquake runs.

	PHASE 1/Infills B In-Plane	PHASE 2/Infills B_FRPU In-Plane (After Emergency Repair)	PHASE 3/Infills B_FRPU Out-Of-Plane (After Specimen Rotation)
INTENSITY	3% KEF-1 (0.06 g)	6% KEF-2 (0.12 g)	6% KEF-3 (0.12 g)
	6% KEF-1 (0.12 g)	11% KEF-2 (0.24 g)	10% KEF-3 (0.22 g)
	11% KEF-1 (0.23g)	18% KEF-2 (0.39 g)	16% KEF-3 (0.35 g)
	25% KEF-1 (0.53g)	–	
	38% KEF-1 (0.80g)		
	55% KEF-1 (1.16g)		
	72% KEF-1 (1.54g)		
	74% KEF-1 (1.57g)		
	69% KEF-1 (1.47g)		
	77% KEF-1 (1.64g)		
